# Supplementary material for: Aggregation pheromones have a non-linear effect on oviposition behavior in Drosophila melanogaster
Source: Nat Commun. 2023 Mar 20;14:1544. doi: 10.1038/s41467-023-37046-2 (PMC10027874; doi:10.1038/s41467-023-37046-2)
Supplement: Supplementary file 1 — Supplementary Information [file 41467_2023_37046_MOESM1_ESM.pdf]

Aggregation pheromones have a non-linear effect on oviposition behavior in *Drosophila melanogaster*

Supplementary information

Thomas A. Verschut[1,2], Renny Ng[3], Nicolas P. Doubovetzky[1], Guillaume Le Calvez[4], Jan L. Sneep[4], Adriaan J. Minnaard[4], Chih-Ying Su[3], Mikael A. Carlsson[2], Bregje Wertheim[1] and Jean-Christophe Billeter[1]

1. Groningen Institute for Evolutionary Life Sciences, University of Groningen, Nijenborgh 7, 9747 AG Groningen, The Netherlands
2. Department of Zoology, Stockholm University, 106 91 Stockholm, Sweden
3. Neurobiology Section, Division of Biological Sciences, University of California, San Diego, La Jolla, CA 92093, USA
4. Stratingh Institute for Chemistry, University of Groningen, Nijenborgh 7, 9747 AG Groningen, The Netherlands

Corresponding Author:
Jean-Christophe Billeter - j.c.billeter@rug.nl

Table S1. Cuticular pheromone profiles of single five days old w^{1118} males housed in groups of one, two, six or twelve individuals and pheromone deposit extracts of groups of males that were housed in a vial for 90 minutes. All reported values are the average concentrations of the compound ± standard error of the mean in ng.

	**Cuticular extract of males housed in groups**				**Pheromone deposit extract of groups of males**			
**Compound**	**1 Male (n=9)**	**2 Males (n=8)**	**6 Males (n=7)**	**12 Males (n=8)**	**1 Male (n=9)**	**2 Males (n=9)**	**6 Males (n=8)**	**12 Males (n=10)**
cVA	123.89 ± 28.48	143.72 ± 42.56	165.88 ± 36.24	94.13 ± 20.18	77.53 ± 32.46	114.08 ± 24.16	213.86 ± 45.28	385.67 ± 96.07
C21:1(7)	52.14 ± 2.24	55.81± 2.63	54.13 ± 1.80	54.36 ± 1.87	24.7 5 ± 0.80	26.74 ± 2.08	31.71 ± 1.02	38.38 ± 3.12
nC22	71.56 ± 5.11	63.69 ± 4.55	55.40 ± 3.75	65.09 ± 7.03	82.26 ± 4.28	84.17 ± 7.22	91.10 ± 4.67	114.62 ± 9.60
C23:1(9)	170.64 ± 13.15	193.47 ± 18.58	165.40 ± 12.67	178.62 ± 14.37	25.98 ± 4.20	49.33 ± 10.56	91.75 ± 9.80	130.73 ± 20.12
7-T	1683.01 ± 125.27	1969.27 ± 196.39	1776.39 ± 130.59	1791.96 ± 136.81	267.64 ± 44.55	497.30 ± 103.22	917.10 ± 101.38	1452.18 ± 157.41
C23:1(5)	138.25 ± 8.72	155.60 ± 14.55	149.49 ± 15.89	147.20 ± 9.39	22.25 ± 4.04	40.80 ± 8.46	78.74 ± 9.48	121.04 ± 12.36
nC23	718.30 ± 29.81	725.57 ± 39.99	698.36 ± 24.35	729.97 ± 36.79	156.50 ± 14.58	231.21 ± 34.16	383.19 ± 30.83	600.74 ± 62.63
C24:1(9)	20.98 ± 1.08	23.42 ± 2.77	19.86± 1.74	21.77 ± 2.22	17.04 ± 1.63	18.27 ± 3.13	22.0 1± 2.17	37.40 ± 4.79
C24:1(5)	10.47 ± 0.91	9.78 ± 1.87	6.64 ± 1.87	7.90 ± 2.46	0 ± 0	0.41 ± 0.41	8.84 ± 2.22	17.19 ± 1.97
nC24	43.68 ± 3.65	36.00 ± 3.33	36.21 ± 7.34	39.77 ± 5.20	0 ± 0	0 ± 0	4.29 ± 1.84	5.79 ± 1.51
2meC24	140.06 ± 25.20	140.13 ± 11.84	124.94 ± 16.76	147.62 ± 20.73	17.45 ± 2.84	32.63 ± 6.58	64.84 ± 9.87	94.49 ± 10.41
C25:1(9)	80.96 ± 3.39	89.23 ± 9.54	71.53 ± 4.66	83.54 ± 7.06	11.57 ± 1.96	21.12 ± 2.72	38.43 ± 4.25	67.72 ± 7.22
C25:1(7)	434.21 ± 21.59	460.20 ± 55.50	360.33 ± 29.93	419.59 ± 46.90	66.54 ± 10.40	111.54 ± 21.14	195.69 ± 25.52	345.35 ± 39.82
C25:1(5)	6.61 ± 1.92	6.03 ± 1.58	0.95 ± 0.95	5.95 ± 1.71	0 ± 0	2.02 ± 2.02	1.69 ± 0.84	5.04 ± 1.55
nC25	76.99 ± 2.32	75.73 ± 4.15	73.50 ± 3.61	78.70 ± 3.03	44.5 8± 1.30	49.97 ± 2.92	66.50 ± 2.63	79.98 ± 7.72
2meC26	113.58± 8.72	119.17 ± 10.78	112.93 ± 7.37	118.74 ± 8.51	15.65 ± 2.50	27.84 ± 4.63	56.76 ± 7.42	89.88 ± 8.93
nC29	13.41 ± 1.08	12.12 ± 3.32	17.00 ± 2.67	18.53 ± 2.07	0 ± 0	0.46 ± 0.46	5.99 ± 1.33	13.60 ± 1.85
**Total**	**3898.73 ± 215.62**	**4278.95 ± 343.25**	**3888.94 ± 221.97**	**4003.43 ± 282.65**	**829.77 ± 100.45**	**1307.88 ± 213.80**	**2272.52 ± 229.15**	**3599.82 ± 419.10**

Table S2. Summary of the total number of eggs ± standard error of the mean (SEM) and the two-tailed Wilcoxon signed rank tests for the pheromone extracts equivalent to the indicated resident group sizes shown in Fig. 1.

			**Males**			**Mated Females**		
**Figure**	**Extract**	**Substrate**	**Eggs ± SEM**	***Z***	***P***	**Eggs ± SEM**	***Z***	***P***
1C	1/8	Sucrose	21.85 ± 1.64	84.5	0.983	21.80 ± 1.13	88	0.931
	1/4	Sucrose	21.80 ± 1.22	146.5	**0.040**	19.60 ± 1.44	146	**0.042**
	1/2	Sucrose	22.30 ± 1.34	191	**0.002**	23.45 ± 1.32	191	**0.001**
	1	Sucrose	22.30 ± 1.86	160.5	**0.009**	19.35 ± 1.67	163.5	**0.030**
	2	Sucrose	20.05 ± 1.28	197	**<0.001**	22.05 ± 1.31	195	**<0.001**
	3	Sucrose	24.35 ± 1.78	183	**<0.001**	20.30 ± 1.18	200	**<0.001**
	4	Sucrose	20.70 ± 1.33	135	0.112	19.60 ± 1.33	160	**0.042**
	5	Sucrose	21.15 ± 1.54	88.5	0.809	22.10 ± 1.55	106	0.673
	6	Sucrose	22.05 ± 1.79	92	0.919	18.55 ± 1.30	82	0.615
	12	Sucrose	21.05 ± 1.39	114	0.751	23.55 ± 1.56	84	0.444
1D	1/8	Sucrose + Yeast	23.35 ± 1.76	147.5	**0.036**	22.05 ± 1.52	155.5	**0.015**
	1/4	Sucrose + Yeast	19.55 ± 1.66	163	**0.032**	21.90 ± 1.69	159	**0.045**
	1/2	Sucrose + Yeast	24.45 ± 2.03	135	**0.033**	20.35 ± 1.83	132.5	**0.042**
	1	Sucrose + Yeast	19.80 ± 0.99	101	0.896	18.25 ± 1.50	178	**0.007**
	2	Sucrose + Yeast	21.80 ± 1.69	72.5	0.586	18.50 ± 1.29	171.5	**0.014**
	3	Sucrose + Yeast	19.95 ± 1.76	90	0.856	17.25 ± 1.91	122.5	0.277
	4	Sucrose + Yeast	21.80 ± 1.40	58	0.394	21.70 ± 1.80	116	0.409
	5	Sucrose + Yeast	20.00 ± 1.84	64	0.219	17.55 ± 1.66	107.5	0.629
	6	Sucrose + Yeast	21.15 ± 1.80	38	**0.041**	16.40 ± 1.18	106	0.985
	12	Sucrose + Yeast	20.45 ± 1.53	34.5	**0.016**	19.15 ± 1.88	92	0.919

Table S3. Summary of the likelihood ratio tests using quasibinomial generalized linear regression models (GLM) or generalized additive regression models (GAM) with the oviposition indices as independent variable, the applicable explanatory variables and appropriate interactions for the comparisons shown in Fig. 1. For GLM and GAM model comparison, the difference in degrees of freedoms *(df)* is reported as Δ_{df}. For the sucrose + yeast substrate the outcome of GLMs for the male and mated female extracts are given separately below the explanatory variable. When applicable the results of a Tukey HSD post-hoc test is given under the explanatory variable in italic.

		**GLM comparing sucrose substrates with sucrose + yeast substrates**		
**Figure**	**Explanatory variable**	***df***	***F***	***P***
1C-D	Sex	1,795	7.43	**<0.001**
	Substrate	1,795	19.32	**<0.001**
	Resident group size	1,795	38.03	**<0.001**
	Sex *x* Substrate	1,795	6.41	**0.011**

		**GAM on sucrose substrate**		
		GLM vs. GAM: Δ_{df} = 4.69, Dev = 137.12, P < 0.001		
**Figure**	**Explanatory variable**	***df***	***F***	***P***
1C	Sex	1,391	0.04	0.840
	Male resident group size	1,391	6.52	**<0.001**
	Female resident group size	1,391	6.05	**<0.001**

		**GLM on sucrose + yeast substrate**		
		GLM vs. GAM: Δ_{df} = 0.59, Dev = 3.57, P = 0.099		
**Figure**	**Explanatory variable**	***df***	***F***	***P***
1D	Sex	1,396	18.28	**<0.001**
	Resident group size	1,396	30.16	**<0.001**
	Sex *x* Resident group size	1,396	1.84	0.175
	Male resident group size	1,198	23.87	**<0.001**
	Female resident group size	1,198	7.99	**0.005**

Table S4. Summary of the total number of eggs ± standard error of the mean (SEM) and the two-tailed Wilcoxon signed rank tests for the pheromone extracts of w^{1118} flies (WT), oenocyte ablated (Oe^-), oenocyte control (Oe^c), or mated females of the indicated genotypes shown in Fig. 2 and S1.

		Males			Females		
**Figure**	**Extract**	**Eggs ± SEM**	***Z***	***P***	**Eggs ± SEM**	***Z***	***P***
2B	Oe^-	20.80 ± 1.13	84.5	0.687	22.45 ± 0.95	45.5	0.085
	♀ Oe^- x ♂ Oe^-	-	-	-	19.60 ± 1.44	100	0.856
	♀ Oe^- x ♂ w^{1118}	-	-	-	20.20 ± 1.52	157.5	**0.013**
	w^{1118}	22.30 ± 1.86	160.5	**0.009**	20.60 ± 1.68	86	0.732
	♀ w^{1118} x ♂ Oe^-	-	-	-	16.20 ± 1.19	72.5	0.868
	♀ w^{1118} x ♂ w^{1118}	-	-	-	21.45 ± 1.86	176.5	**0.008**
S1B	Oe^c (virgin)	20.70 ± 1.43	168.5	0.019	22.70 ± 1.25	95	1.000
	♀ Oe^c x ♂ Oe^-	-	-	-	18.30 ± 1.66	57.5	0.381
	♀ Oe^c x ♂ Oe^c	-	-	-	19.30 ± 1.74	141.5	0.064
	♀ Oe^c x ♂ w^{1118}	-	-	-	20.00 ± 1.43	167.5	**0.021**
	♀ Oe^- x ♂ Oe^c	-	-	-	22.00 ± 1.66	128.5	0.184
	♀ w^{1118} x ♂ Oe^c	-	-	-	18.35 ± 1.77	133.5	**0.038**

Table S5. Summary of the likelihood ratio tests using quasibinomial generalized linear regression models (GLM) with the oviposition indices as independent variable and the applicable explanatory variables for the comparisons shown in Fig. 2 and S1.

		**GLM comparing fly pheromone extracts**		
**Figure**	**Explanatory variable**	***df***	***F***	***P***
2B	Oe$^-$ virgin female *vs.* Oe$^-$ male	1,38	0.81	0.374
	Oe$^-$ *x* Oe$^-$ *vs.* Oe$^-$ *x* w^{1118}	1,38	2.04	0.161
	w^{1118} virgin female *vs.* w^{1118} male	1,38	4.79	**0.035**
	w^{1118} *x* Oe$^-$ *vs.* w^{1118} *x* w^{1118}	1,38	7.27	**0.010**
		**GLM and Tukey HSD comparing mated female extracts**		
**Figure**	**Explanatory variable**	***df***	***F***	***P***
S1B	Oe$^-$ virgin female *vs.* Oe$^-$ male	1,38	3.59	0.066
	Oe^c mated female genotypes	1,38	4.31	0.116
	Oe$^-$ *x* Oe^c *vs.* w^{1118} *x* Oe^c	1,38	0.162	0.689

Table S6. Summary of the total number of eggs ± standard error of the mean (SEM) and the two-tailed Wilcoxon signed rank tests for the pheromones shown in Fig. 2 and S1. For cis-11-Vaccenyl Acetate (cVA), 7-Tricosene (7-T), 9-Tricosene (9-T), 7,11-Heptacosadiene (7,11-HD) the concentrations in nanograms (ng) refer to two, six and twelve times the fly pheromone equivalents. Concentrations separated by a '+' refer to the concentration of cVA followed by that of the indicated compound.

			CHC alone			cVA + CHC		
**Figure**	**Treatment**	**Concentration**	**Eggs ± SEM**	***Z***	***P***	**Eggs ± SEM**	***Z***	***P***
2C	cVA	560	-	-	-	21.45 ± 1.68	114.5	0.444
		1680	-	-	-	22.40 ± 1.64	113	0.779
		3360	-	-	-	22.05 ± 1.76	61	0.477
	7-T	560 + 280	19.55 ± 1.63	96	0.984	20.45 ± 1.21	201	**<0.001**
		1680 + 840	21.70 ± 1.42	104.5	0.421	21.55 ± 1.79	195	**<0.001**
		3360 + 1680	20.80 ± 1.36	72	0.225	21.15 ± 1.43	128	0.401
	9-T	560 + 28	20.25 ± 1.44	99.5	0.852	19.00 ± 1.06	155.5	**0.016**
		1680 + 84	23.65 ± 1.59	91	0.614	18.40 ± 1.53	107.5	0.629
		3360 + 168	20.75 ± 1.22	119	0.614	20.45 ± 1.34	127	0.422
	7,11-HD	560 + 620	21.05 ± 1.37	111.5	0.823	23.55 ± 1.27	126	0.219
		1680 + 1860	21.70 ± 1.27	89.5	0.879	23.10 ± 1.53	126.5	0.212
		3360 + 3720	22.20 ± 1.49	99.5	0.852	21.65 ± 1.72	101	0.513
S1C	9-T	560 + 280	-	-	-	17.75 ± 1.62	163.5	**0.006**
		1680 + 840	-	-	-	17.45 ± 1.18	118	0.641
		3360 + 1680	-	-	-	17.90 ± 1.63	110	0.559

Table S7. Summary of the likelihood ratio tests using quasibinomial generalized linear regression models (GLM) with the oviposition indices as independent variable and the applicable explanatory variables for the comparisons shown in Fig. 2. When applicable the results of a Tukey HSD post-hoc test is given under the explanatory variable in italic. Concentrations in nanograms (ng) refer to two, six and twelve times the fly pheromone equivalents.

		**GLMs comparing pheromone concentrations**		
**Figure**	**Explanatory variable**	***df***	***F***	***P***
2C	cVA concentration	1,57	0.77	0.381
	7-T concentration	1,57	0.97	0.324
	9-T concentration	1,57	0.34	0.561
	7,11-HD concentration	1,57	0.149	0.699
	cVA + 7-T concentrations	1,57	6.83	**0.009**
	560 + 280 vs. 1680 + 840	-	-0.42	0.909
	560 + 280 vs. 3360 + 1680	-	-2.56	**0.028**
	1680 + 840 vs. 3360 + 1680	-	-2.15	0.079
	cVA + 9-T concentrations	1,57	1.56	0.282
	cVA + 7,11-HD concentrations	1,57	0.43	0.514

Table S8. Summary of the total number of eggs ± standard error of the mean (SEM) and the two-tailed Wilcoxon signed rank tests for the pheromones shown in Fig. 3 and S1. For heptanal, hexadecanal, heptanoic acid and palmitic acid the concentrations refer to two, six and twelve times the 7-T pheromone equivalents. Concentrations separated by a '+' refer to the concentration of cVA followed by that of the indicated compound.

			**Oxidation product**			**cVA + Oxidation product**		
**Figure**	**Treatment**	**Concentration**	**Eggs ± SEM**	***Z***	***P***	**Eggs ± SEM**	***Z***	***P***
3C - S1E	Heptanal	560 + 100	18.40 ± 1.24	83.5	0.658	20.20 ± 1.49	171	**0.014**
		1680 + 300	18.95 ± 1.51	108.5	0.601	19.85 ± 1.63	173	**0.012**
		3360 + 600	16.20 ± 1.37	63	0.338	20.50 ± 1.40	80	0.828
	Hexadecanal	560 + 210	19.85 ± 1.63	89	0.571	17.60 ± 1.49	98.5	0.586
		1680 + 630	18.30 ± 1.55	115	0.432	19.20 ± 1.65	99	0.571
		3360 + 1260	17.80 ± 1.21	86	0.670	14.45 ± 1.10	74	0.409
	Heptanoic acid	560 + 115	16.90 ± 1.34	69.5	0.758	16.30 ± 1.44	95.5	0.737
		1680 + 345	16.70 ± 1.41	57	0.225	18.05 ± 1.45	115.5	0.421
		3360 + 690	16.40 ± 1.06	87	0.965	17.00 ± 1.32	100	0.867
	Palmitic acid	560 + 220	18.90 ± 1.54	78	0.761	21.10 ± 1.61	63	0.121
		1680 + 660	19.75 ± 1.66	111	0.533	17.45 ± 1.59	91	0.614
		3360 + 1320	17.15 ± 0.78	106.5	0.372	19.35 ± 1.61	72.5	0.376
S1F	cVA + Heptanal	560 + 27	20.75 ± 1.17	74	0.632	20.05 ± 1.18	203	**0.001**
		1680 + 81	21.00 ± 1.26	101	0.825	19.6 ± 1.37	192	**0.001**
		3360 + 162	21.25 ± 1.21	111.5	0.823	19.65 ± 1.48	130	0.361
		560 + 270	21.15 ± 1.58	123.5	0.259	20.75 ± 1.19	203	**<0.001**
		1680 + 810	22.65 ± 1.14	129	0.177	24.10 ± 1.48	192	**0.001**
		3360 + 1620	25.90 ± 1.48	103	0.763	24.65 ± 1.22	130	0.361

Table S9. Summary of the likelihood ratio tests using quasibinomial generalized linear regression models (GLM) with the oviposition indices as independent variable and the applicable explanatory variables for the comparisons shown in Fig. 3 and S1. When applicable the results of a Tukey HSD post-hoc test is given under the explanatory variable in italic. Concentrations in nanograms (ng) refer to two, six and twelve times the fly pheromone equivalents. Concentrations separated by a '+' refer to the concentration of cVA followed by that of the indicated compound.

		GLM comparing cVA + oxidation product concentrations		
**Figure**	**Explanatory variable**	***df***	***F***	***P***
3C	cVA + heptanal	1,57	4.69	**0.031**
	560 + 100 vs. 1680 + 300	-	0.63	0.998
	560 + 100 vs. 3360 + 600	-	-2.16	0.079
	1680 + 300 vs. 3360 + 600	-	-2.22	0.068
	cVA + hexadecanal	1,57	1.48	0.224
	cVA + heptanoic acid	1,57	0.02	0.881
	cVA + palmitic acid	1,57	0.11	0.740

		GLMs comparing oxidation products		
**Figure**	**Explanatory variable**	***df***	***F***	***P***
S1E	Heptanal	1,57	0.10	0.755
	Hexadecanal	1,57	0.19	0.656
	Heptanoic acid	1,57	0.56	0.454
	Palmitic acid	1,57	0.78	0.376

		GLM comparing heptanal concentrations		
**Figure**	**Explanatory variable**	***df***	***F***	***P***
S1F	Heptanal (low)	1,57	0.35	0.706
	cVA + Heptanal (low)	1,57	4.11	**0.022**
	Heptanal (high)	1,57	0.29	0.750
	cVA + Heptanal (high)	1,57	3.70	**0.031**

Table S10. Summary of the total number of eggs ± standard error of the mean (SEM) and the two-tailed Wilcoxon signed rank tests for the combinations of cVA with 7-T or heptanal shown in Fig. 3. Concentrations separated by a '+' refer to the concentration of cVA followed by that of the indicated compound.

	**cVA + 7-T**				**cVA + Heptanal**			
**Figure**	**Concentration**	**Eggs ± SEM**	***Z***	***P***	**Concentration**	**Eggs ± SEM**	***Z***	***P***
3D	560 + 280	21.25 ± 1.47	181	**0.003**	560 + 100	20.20 ± 1.49	171	**0.014**
	560 + 840	20.70 ± 1.38	174.5	**0.009**	560 + 300	18.35 ± 1.54	168	**0.003**
	560 + 1680	20.35 ± 1.23	179	**0.006**	560 + 600	16.05 ± 1.34	168.5	**0.019**
	1680 + 280	19.65 ± 1.30	136	**0.029**	1680 + 100	18.90 ± 1.66	147	**0.038**
	1680 + 840	22.05 ± 1.29	152	**0.023**	1680 + 300	19.85 ± 1.63	173	**0.012**
	1680 + 1680	19.40 ± 1.47	118.5	0.355	1680 + 600	20.20 ± 1.67	166.5	**0.023**
	3360 + 280	18.80 ± 1.03	70	0.197	3360 + 100	18.85 ± 1.44	66	0.408
	3360 + 840	19.50 ± 1.52	122.5	0.277	3360 + 300	17.35 ± 1.52	92.5	0.777
	3360 + 1980	19.10 ± 1.51	115	0.723	3360 + 600	20.50 ± 1.40	80	0.828

Table S11. Summary of the likelihood ratio tests using quasibinomial generalized linear regression models (GLM) with the oviposition indices as independent variable and the applicable explanatory variables and appropriate interactions for the comparisons shown in Fig. 3. Explanatory variables with *P* values above 0.05 are reported if backward selection did not improve the model fit. When applicable the results of a Tukey HSD post-hoc test is given under the explanatory variable in italic.

		**GLMs comparing cVA + 7-T and cVA + Heptanal all doses**		
**Figure**	**Explanatory variable**	***df***	***F***	***P***
3D	Dose	1,356	31.65	**<0.001**
	Odor	1,356	0.37	0.543
	Dose *x* Odor	1,356	0.06	0.807
	560 vs. 1680	-	-1.86	0.151
	560 vs. 3360	-	-5.53	**<0.001**
	1680 vs. 3360	-	-3.81	**<0.001**
		**GLMs comparing cVA + 7-T and cVA + Heptanal at 560 ng**		
**Figure**	**Explanatory variable**	***df***	***F***	***P***
3D	Odor	1,117	0.43	0.512
	Concentration	1,117	0.14	0.712
	Odor *x* Concentration	1,117	0.03	0.873
		**GLMs comparing cVA + 7-T and cVA + Heptanal at 1680 ng**		
**Figure**	**Explanatory variable**	***df***	***F***	***P***
3D	Odor	1,117	0.24	0.629
	Concentration	1,117	1.38	0.242
	Odor *x* Concentration	1,117	0.73	0.395
		**GLMs comparing cVA + 7-T and cVA + Heptanal at 3360 ng**		
**Figure**	**Explanatory variable**	***df***	***F***	***P***
3D	Odor	1,117	0.69	0.401
	Concentration	1,117	0.71	0.400
	Odor *x* Concentration	1,117	0.05	0.817

Table S12. Summary of the total number of eggs ± standard error of the mean (SEM) and the two-tailed Wilcoxon signed rank tests for all genotypes per combination of *cis*-11-Vaccenyl Acetate (cVA) + 7-Tricosene (7-T) in nanograms (ng) that refer to two, six and twelve times the fly pheromone equivalents as shown in Fig. 4. The w^{1118} cross column indicates the results of the control flies and the UAS-Kir2.1 cross column indicates the results of flies with silenced receptor neurons.

			**w^{1118} cross**			**UAS-Kir2.1 cross**		
Figure	***Receptor***	***cVA + 7-T***	***Eggs ±SEM***	***Z***	***P***	***Eggs ±SEM***	***Z***	***P***
Fig. 4A	w^{1118}	560 + 280	20.45 ± 1.21	201	**<0.001**	24.55 ± 2.13	152.5	**0.022**
		1680 + 840	21.55 ± 1.79	195	**<0.001**	27.75 ± 1.96	184	**0.003**
		3360 + 1680	21.15 ± 1.43	128	0.401	28.20 ± 1.59	101	0.514
Fig. 4B	Orco	560 + 280	21.45 ± 1.79	170	**0.002**	33.60 ± 2.59	66	0.154
		1680 + 840	20.70 ± 1.63	160	**0.042**	32.85 ± 2.65	123	0.514
		3360 + 1680	21.50 ± 2.25	10	0.644	35.85 ± 2.65	94	0.984
Fig. 4C	Or65a	560 + 280	25.40 ± 1.68	192	**0.001**	24.65 ± 1.58	194	**<0.001**
		1680 + 840	23.30 ± 1.78	167	**0.022**	18.15 ± 1.28	104.5	0.717
		3360 + 1680	23.45 ± 1.98	107	0.644	22.90 ± 1.65	69	0.305
Fig. 4C	Or67d	560 + 280	25.30 ± 1.76	172	**0.013**	26.55 ± 1.43	56.5	0.126
		1680 + 840	22.35 ± 1.78	148	**0.035**	26.10 ± 1.86	204	**<0.001**
		3360 + 1680	22.60 ± 1.37	104.5	0.421	26.00 ± 1.89	147.5	**0.036**
Fig. 4D	Gr32a	560 + 280	31.50 ± 1.67	209	**<0.001**	33.45 ± 1.89	209	**<0.001**
		1680 + 840	27.85 ± 1.86	178	**0.005**	26.35 ± 1.87	143	0.165
		3360 + 1680	26.80 ± 1.52	102	0.927	25.00 ± 1.43	86	0.732
Fig. 4E	Or13a	560 + 280	25.30 ± 1.51	194	**<0.001**	22.20 ± 1.26	144.5	0.145
		1680 + 840	22.60 ± 1.49	170.5	**0.015**	21.45 ± 1.42	151	0.089
		3360 + 1680	24.75 ± 1.66	91.5	0.904	19.75 ± 1.46	106	0.985
Fig. 4E	Or22a	560 + 280	25.30 ± 1.58	170.5	**0.015**	22.80 ± 1.87	85.5	0.478
		1680 + 840	25.50 ± 1.97	165	**0.024**	20.60 ± 1.88	109.5	0.306
		3360 + 1680	24.15 ± 2.37	101.5	0.809	18.75 ± 1.67	132	0.142
Fig. 4E	Or35a	560 + 280	30.25 ± 2.13	175	**0.009**	15.00 ± 1.39	140.5	0.191
		1680 + 840	30.15 ± 2.18	144.5	**0.049**	17.65 ± 1.92	117	0.387
		3360 + 1680	32.05 ± 2.01	105.5	0.999	16.80 ± 1.46	58	0.142
Fig. 4E	Or67b	560 + 280	19.75 ± 1.83	177	**0.008**	22.05 ± 1.93	77	0.728
		1680 + 840	21.65 ± 1.59	158	**0.049**	22.55 ± 1.94	125.5	0.085
		3360 + 1680	28.25 ± 2.05	65	0.235	21.30 ± 1.99	87	0.636
Fig. 4E	Or69a	560 + 280	27.85 ± 2.34	191	**0.001**	19.85 ± 1.77	89	0.825
		1680 + 840	29.20 ± 2.09	170	**0.016**	23.50 ± 1.25	158	**0.049**
		3360 + 1680	22.95 ± 1.81	108	0.615	22.05 ± 1.59	164	**0.005**

Table S13. Summary of the likelihood ratio tests using quasibinomial generalized linear regression models (GLM) or generalized additive regression models (GAM) with the oviposition indices as independent variable and the applicable explanatory variables and appropriate interactions for the comparisons shown in Fig. 4. Explanatory variables with *P* values above 0.05 are reported if backward selection did not improve the model fit. Receptors for which the GAM outcompeted the GLM have the outcome of the non-linear analysis reported. If the model comparison did not result in a $\Delta_{df} \geq 0.00$ only the GLM outcome is given. When applicable the outcome of GLMs for the control and silenced crosses are given separately below the Genotype x Concentration explanatory variable. The concentrations in nanograms (ng) refer to two, six and twelve times the fly pheromone equivalents. When applicable the results of a Tukey HSD post-hoc test is given under the explanatory variable in italic.

		GLM on w^{1118}		
**Figure**	**Explanatory variable**	***df***	***F***	***P***
4A	Genotype	1,116	0.60	0.439
	Concentration	1,116	12.41	**<0.001**
	Genotype x Concentration	1,116	0.06	0.809
	w^{1118}	1,58	7.32	**0.009**
	w^{1118} x Kir2.1	1,58	5.46	**0.023**
		GLM on Orco		
**Figure**	**Explanatory variable**	***df***	***F***	***P***
4B	Genotype	1,116	8.17	**0.008**
	Concentration	1,116	0.35	0.970
	Genotype x Concentration	1,116	4.13	0.057
	Orco x w^{1118}	1,58	2.89	0.095
	Orco x Kir2.1	1,58	1.17	0.284
		GLM on Or65a		
**Figure**	**Explanatory variable**	***df***	***F***	***P***
4C	Genotype	1,116	5.42	0.022
	Concentration	1,116	12.67	**<0.001**
	Genotype x Concentration	1,116	1.79	0.182
	Or65a x w^{1118}	1,58	2.40	0.127
	Or65a x Kir2.1	1,58	12.05	**<0.001**
		GAM on Or67d<br>*GLM vs. GAM: Δdf = 0.94, Dev = 18.48, P < 0.001*		
**Figure**	**Explanatory variable**	***df***	***F***	***P***
4C	Genotype	1.00	1.00	1.00
	Concentration	-	-	-
	Genotype x Concentration	-	-	-
	Or67d x w^{1118}	1.00	1.00	1.00
	Or67d x Kir2.1	1.99	1.99	1.99
		GLM on Gr32a		
**Figure**	**Explanatory variable**	***df***	***F***	***P***
4D	Genotype	1,116	0.729	0.395
	Concentration	1,116	24.25	**<0.001**
	Genotype x Concentration	1,116	0.05	0.827
	Gr32a x w^{1118}	1,58	9.62	**0.003**
	Gr32a x Kir2.1	1,58	15.52	**<0.001**

Table S13. Continued.

		GLM on Or13a		
**Figure**	**Explanatory variable**	***df***	***F***	***P***
4E	Genotype	1,116	0.04	0.849
	Concentration	1,116	7.86	**0.006**
	Genotype x Concentration	1,116	1.86	0.175
	*Or13a x w^1118^* → $Or13a \times w^{1118}$	1,58	10.45	**0.002**
	Or13a x Kir2.1	1,58	0.71	0.404
		GLM on Or22a		
**Figure**	**Explanatory variable**	***df***	***F***	***P***
4E	Genotype	1,116	2.62	0.108
	Concentration	1,116	0.06	0.815
	Genotype x Concentration	1,116	7.31	**0.008**
	Or22a x w^{1118}	1,58	3.95	**0.050**
	Or2a x Kir2.1	1,58	3.40	0.070
		GLM on Or35a		
**Figure**	**Explanatory variable**	***df***	***F***	***P***
4E	Genotype	1,116	3.89	0.051
	Concentration	1,116	3.98	**0.048**
	Genotype x Concentration	1,116	0.25	0.618
	Or35a x w^{1118}	1,58	2.79	0.100
	Or35a x Kir2.1	1,58	0.85	0.359
		GAM on Or67b *GLM vs. GAM: Δdf = 1.23, Dev = 21.81, P = 0.035*		
**Figure**	**Explanatory variable**	***df***	***df***	***df***
4E	Genotype	114.77	114.77	114.77
	Concentration	-	-	-
	Genotype x Concentration	-	-	-
	Or67b x w^{1118}	1.91	1.91	1.91
	Or67b x Kir2.1	1.78	1.78	1.78
		GLM on Or69a		
**Figure**	**Explanatory variable**	***df***	***F***	***P***
4E	Genotype	1,116	2.44	0.121
	Concentration	1,116	0.10	0.776
	Genotype x Concentration	1,116	8.36	**0.005**
	Or69a x w^{1118}	1,58	4.11	**0.047**
	Or69a x Kir2.1	1,58	4.31	**0.042**

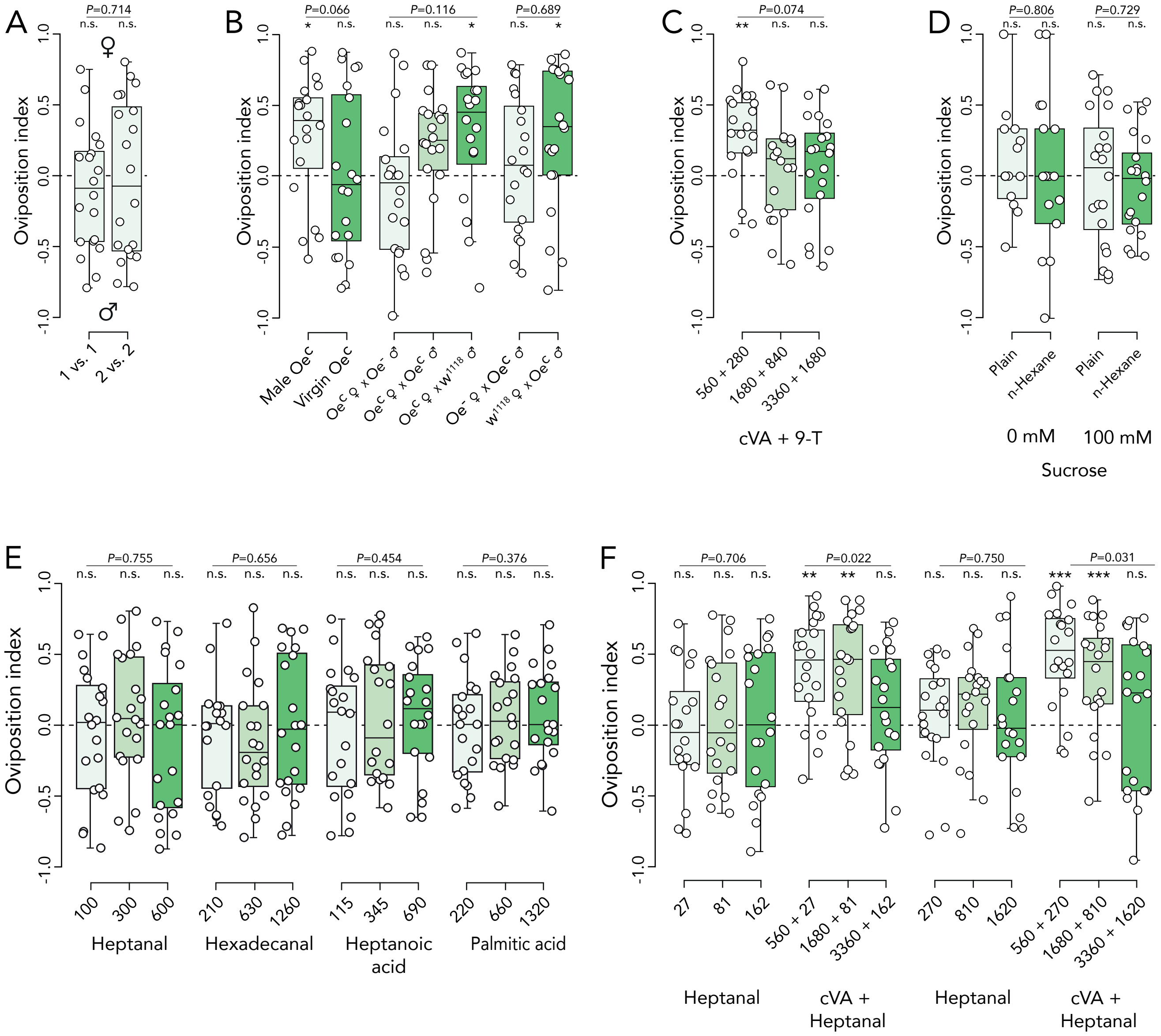

Figure S1. Extracts and pheromones. (**A**) Oviposition preference in a two-choice test between the pheromone extracts of one and two mated females (1.00) versus one or two males (-1.00) (GLM: $F_{1,38}$= 0.14, P = 0.714). (**B**) Oviposition preference to extracts of oenocyte control flies (Oe^c) and extracts of mated females which were acquired by mating a female with a male the indicated genotypes (oenocyte ablated (Oe$^-$)). (**C**) Oviposition preference to 10-times higher doses of 9-Tricosene pheromone equivalents (9-T: 280 - 840 - 1680 ng) in combination with cVA. The concentrations were tested to match the pheromone equivalents of 7-T (for which we found an effect), but no dose-dependent decrease occurred after increasing the dose of 9-T tenfold (GLM: $F_{1,57}$ = 5.21, P = 0.074). (**D**) Oviposition preference on a plain substrate without solvent (mean number of eggs 3.90 ± 0.78) and *n*-Hexane (3.95 ± 0.73) when combined with 0 mM sucrose (GLM: $F_{1,38}$= 0.06, P = 0.806) and to a plain substrate (13.00 ± 1.37) and *n*-Hexane (22.30 ± 1.54) when combined with agar containing 100 mM of sucrose (GLM: $F_{1,38}$= 0.12, P = 0.729). (**E**) Oviposition preference to two, six and twelve times the pheromone oxidation equivalent of heptanal (100 - 300 - 600 ng; GLM: $F_{1,57}$ = 0.10, P = 0.755), hexadecanal (210 - 630 - 1260 ng; GLM: $F_{1,57}$ = 0.19, P = 0.656), heptanoic acid (115 - 345 - 690 ng; GLM: $F_{1,57}$ = 0.56, P = 0.454) and palmitic acid (220 - 660 - 1320 ng; GLM: $F_{1,57}$ = 0.78, P = 0.376). (**F**) Oviposition preference to two, six and twelve times the pheromone equivalent of heptanal and combination of heptanal with cVA based on the percentage of aldehydes found in the headspace of flies as reported by Lebreton *et al.* (2017) BMC Biol. 15:88, followed by tenfold multiplications of those doses. Changing the concentration of heptanal has no effect on the dose-dependent response. Asterisks above each treatment indicate whether preference differs significantly from zero (indicating no preference) as determined by a two-tailed Wilcoxon signed rank tests. Difference in preference across treatments was tested with a GLM with quasibinomial error distribution and is indicated above the bar (n=20 for all treatments). The center line of the box plots denotes the median value (50th percentile), the box contains the 25th to 75th percentiles of dataset. The black whiskers mark 1.5 times the interquartile range. * P < 0.05; ** P < 0.01; *** P < 0.001. n.s. not significant. Source data are provided as a Source Data file

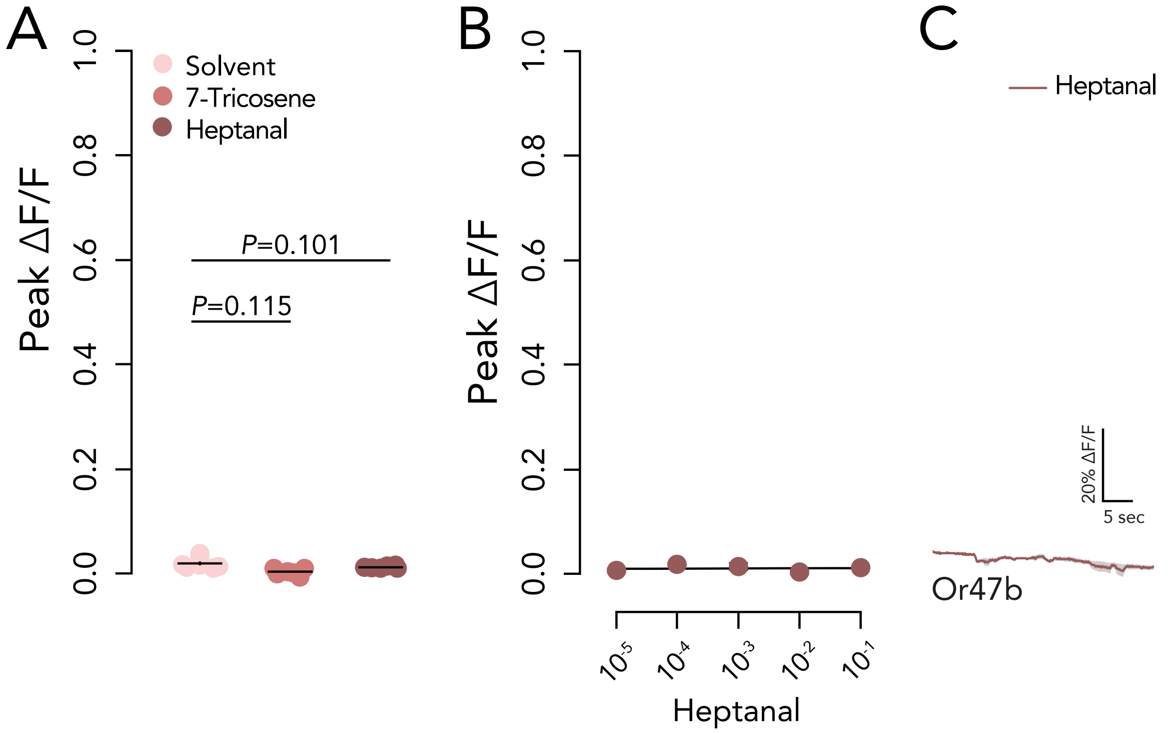

Figure S2. Transcuticular calcium imaging of Or47b olfactory receptor neurons as negative control. (**A**) Quantification (mean ± standard error of the mean) of the transcuticular calcium responses from Or47b to the paraffin oil solvent (n=7), 7-Tricosene (n=6), and heptanal (n=7). The indicated statistical differences between the treatments were calculated with two-tailed t-tests comparing the responses to the solvent and 7-Tricosene and the solvent and heptanal (10^{-2} dilution). (**B**) Dose-response curves of Or47b to aliquots of heptanal (n=7 per aliquot). The error bars indicate the standard error of the mean. (**C**) Calcium fluorescence traces of Or47b in response to a 10^{-2} dilution of heptanal visualized with the standard error of the mean as grey shaded areas. Source data are provided as a Source Data file.
